# No evidence for a link between forest herbicides and offspring sex ratio in a migratory songbird using high-throughput molecular sexing

**DOI:** 10.1093/conphys/cox054

**Published:** 2017-09-22

**Authors:** James W Rivers, Jennifer L Houtz, Matthew G Betts, Brent M Horton

**Affiliations:** 1 Department of Forest Ecosystems and Society, Oregon State University, Corvallis, OR 97331, USA; 2 Department of Biology, Millersville University, Millersville, PA 17551, USA; 3 Forest Biodiversity Research Network, Department of Forest Ecosystems and Society, Oregon State University, Corvallis, OR 97331, USA

**Keywords:** direct PCR, forest herbicides, molecular sexing, offspring sex ratio, White-crowned Sparrow, *Zonotrichia leucophrys*

## Abstract

Many species that use or require early-successional forest are of conservation concern, including a number of songbirds that have experienced long-term population declines. In this study, our initial goal was to test whether herbicide application intensity was linked to offspring sex ratio in the White-crowned Sparrow (*Zonotrichia leucophrys*), a species that requires early-successional forest within forested landscapes. However, a rapid and accurate method using direct PCR to sex a large sample of birds (*n* > 1000 individuals) was unavailable, so our secondary goal was to develop a new approach for rapidly determine offspring sex. We obtained blood samples from sparrow young during the 2013–2014 breeding seasons in regenerating conifer plantations that were treated with one of four treatments (i.e. light, moderate, and intensive herbicide application, or no-spray control). We then optimized a protocol that used a commercially available, direct PCR kit to amplify sex-specific fragments of the CHD (chromo-helicase-DNA-binding) genes directly from whole blood stored in lysis buffer. Using this approach, we found no evidence that offspring sex ratio was linked to herbicide application intensity or to food availability across herbicide treatments. Our molecular sexing technique was 100% accurate when validated on known-sex adults, and 99.9% of our blood samples amplified successfully after being stored in lysis buffer stored for up to 3 years. The application of direct PCR for sexing birds eliminated the need for DNA extraction and substantially reduced sample processing time, cost, and the opportunity for errors during the extraction step. We conclude that forest herbicide application intensity does not influence sparrow offspring sex ratio in our study system, and that our approach provides a rapid, accurate, and tractable method for sexing birds that can facilitate studies that require processing of a large number of samples.

## Teaser

Forest management practices can influence offspring sex ratio in birds. Our large-scale experimental study developed a high-throughput approach to determine the sex of >1000 songbird nestlings, and in using it we found no evidence that offspring sex ratio varied as a function of herbicide application intensity.

## Introduction

Concern has increased in recent years regarding species that require early-successional forest habitat due to long-term population declines (Litvaitis, 1993; Thompson and DeGraaf, 2001; Schlossberg and King, 2009; King *et al.*, 2011; Swanson *et al.*, 2011; King and Schlossberg, 2014; Kwit *et al.*, 2014), including songbirds that require broadleaf vegetation for foraging and nesting during the breeding season (Hagar, 2007; Betts *et al.*, 2010; Ellis and Betts, 2011). Indeed, a recent review found that >50% of species of conservation concern in the Pacific Northwest region of North America use or require early-successional forest during their life cycle, including many state conservation-listed species (Swanson *et al.*, 2014). Population declines of early-successional forest species have been attributed to the scarcity of this forest type on the landscape (Kennedy and Spies, 2005; Thomas *et al.*, 2006; King and Schlossberg, 2014), as well as contemporary forest management practices, especially within intensively managed timber plantations. Such practices can include the use of forest herbicides to control undesirable vegetation (Wagner *et al.*, 2004; Hayes *et al.* 2005; Maguire *et al.*, 2009; Jokela *et al.*, 2010) and increase the growth of commercial species (Cole and Newton, 1987; Wagner *et al.*, 2004; Maguire *et al.*, 2009). Forest herbicides have the potential to negatively impact animal populations through direct toxic effects, which can be acute and result in mortality, in addition to indirect effects, which exert subtler effects yet still can have longer-term impacts on populations (Kohler and Triesbkorn, 2013). To date there has been limited evidence for direct toxic effects of forest herbicides on free-ranging animal populations (Tatum, 2004; McComb *et al.*, 2008; but see Relyea, 2005), and this has been attributed to the short retention time of herbicides (Newton *et al.*, 1984) and their targeting of plant-specific physiological mechanisms (Tatum, 2004; McComb *et al.*, 2008). In contrast, the indirect effects of herbicides are thought to play a more important role in influencing animal populations, most commonly through herbicide-mediated changes to habitat (Betts *et al.*, 2013; Kroll *et al.*, 2017).

Despite their widespread use, we have little information regarding how forest herbicides impact animal demographic processes (Lautenschlager and Sullivan, 2002; Guynn *et al.*, 2004; Lautenschlager and Sullivan, 2004; Kohler and Triebskorn, 2013). This is especially true for species that require early-successional forests (Betts *et al.*, 2010; Swanson *et al.*, 2014), as this forest age class is most likely subjected to herbicide application (Wagner *et al.*, 2004). Previous work on this topic has focused primarily on descriptive surveys to evaluate the indirect effects of forest herbicides on animal populations (Morrison and Meslow, 1984; Santillo *et al.*, 1989; Woodcock *et al.*, 1997), and manipulative studies are lacking that evaluate whether forest herbicides are linked to subtle changes in population characteristics related to breeding productivity, such as the sex ratio of offspring. Adjustments in offspring sex ratio may be an adaptive process by which parents skew sex ratio in a manner that enhances their fitness relative to environmental conditions (Trivers and Willard, 1973; Charnov, 1982). In contrast, herbicides may lead to non-adaptive changes in offspring sex ratio that occur through direct physiological processes, such as endocrine disruption (Willingham, 2005; McKinlay *et al.*, 2008; Erikstad *et al.*, 2011), or indirectly via habitat alterations that reduce critical resources needed for reproduction and increase nutritional stress for females (Myers, 1978). Given that environmentally mediated variation in sex ratio can have a strong influence on the structure and dynamics of animal populations (Mills and Smouse, 1994; Kruuk *et al.*, 1999; Le Galliard *et al.*, 2005), it is important to understand whether songbird offspring sex ratio is influenced by forest herbicide application within intensively managed early-successional forest.

In this study, we used a large-scale experiment to test whether herbicide application intensity was related to offspring sex ratio in the White-crowned Sparrow (*Zonotrichia leucophrys*, hereafter sparrow), a species that requires early-successional forest for breeding habitat within forested landscapes. Broadleaf shrubs, herbs, and grasses in managed forests of the Pacific Northwest typically harbor a greater diversity and abundance of invertebrate prey relative to conifers (e.g. lepidopteran larvae; Hammond and Miller, 1998), and they are often used as foraging substrates by breeding songbirds. Yet, these plant functional groups compete with commercial tree species and are often subjected to forest herbicide application, and this management action has the potential to temporarily reduce songbird food availability during breeding. Poor conditions for rearing young, in turn, can lead to changes in offspring sex ratio (Trivers and Willard, 1973; Pike and Petrie, 2003), with the expectation that females will bias broods towards female offspring as rearing conditions decline (Mead *et al.*, 1987; Nager *et al.*, 1999; Bonier *et al.*, 2007). Thus, the primary objective of our study was to test whether offspring sex ratio of sparrows become increasingly female-biased with increasing herbicide application intensity. Given that food availability can influence offspring sex ratio (Myers, 1978; Nager *et al.*, 1999), we also used arthropod data collected during a related investigation to test whether sex ratio was related to food availability during breeding.

To meet our primary study objective, we required a methodological approach that allowed us to accurately assign sex to a large number of blood samples from nestling sparrows (*n* > 1000) in a time- and cost-efficient manner. The absence of sexual dimorphism in sparrow young, as in many bird species, requires a molecular approach to accurately determine offspring sex (Griffiths *et al.*, 1998; Dubiec and Zagalska-Neubauer, 2006). A common sexing technique used in avian field studies involves using polymerase chain reaction (PCR) to amplify sex-specific genes (e.g. the chromo-helicase-DNA-binding or CHD genes; Griffiths *et al.*, 1998) located on the Z and W sex chromosomes. Although such PCR-based sexing techniques are effective, most require purified DNA for analysis that is obtained through the expensive and time-consuming process of DNA extraction. More recently, direct PCR approaches have been developed that allow for the amplification of DNA fragments directly from blood and other tissues without the need for the DNA extraction step (Swaran, 2014), although this technique has experienced limited application in avian studies (Morinha *et al.*, 2012; but see Tomasulo *et al.*, 2002; Quintana *et al.*, 2009; Suriyaphol *et al.*, 2014; Dhanasekaran *et al.*, 2016). Further development of rapid, accurate and cost-effective direct PCR approaches for sexing birds can increase the tractability of large-scale studies of offspring sex ratio and, more broadly, encourage wider application of molecular sexing in avian studies to identify and account for sex differences in physiology, behavior and life history. Therefore, our second objective was to optimize a tractable direct PCR protocol for the rapid molecular sexing of nestlings from blood samples, and evaluate its accuracy using a sample of known-sex adults.

## Materials and methods

### Study area and species

We undertook this investigation during the 2013–2014 breeding seasons in the western hemlock (*Tsuga heterophylla*) vegetation zone of the Coast Range Mountains in western Oregon, USA (Franklin and Dyrness, 1988). This region is currently dominated by intensively managed Douglas-fir (*Pseudotsuga menziesii*) plantations that often contain broadleaf shrubs (e.g. vine maple [*Acer rubra*], bigleaf maple [*A. macrophyllum*], red elderberry [*Sambucus racemosa*], *Rubus* spp.), evergreen ground cover (e.g. salal [*Gaultheria shallon*], Oregon grape [*Mahonia nervosa*]), and various ferns, grasses and herbaceous forbs that are strong competitors with recently planted commercial tree species.

We used a randomized complete block experimental design, and each of our study blocks (*n* = 4) contained four stands that were each subjected to a distinct treatment (*n* = 16 stands total, four replicate stands per treatment). All stands within each study block were located within 5 km of each other to provide spatially independent treatments while minimizing additional landscape-scale variation. All stands were of typical size for our study region (10–18 ha), harvested in winter 2010, and re-planted with Douglas-fir seedling nursery stock in 2011. Treatments included a no-spray control, as well as a light, moderate, or intensive herbicide application. All herbicide treatments were applied at the stand scale, and the light and moderate herbicide treatments represent the range of operational practices within our study area. Control stands were not subjected to any herbicide application during the course of this study. The light herbicide treatment consisted of a herbaceous spray in 2011 (1 year post-harvest) and a broadleaf release spray in 2012, whereas the moderate herbicide treatment consisted of a site preparation broadleaf spray in 2010 prior to planting, followed by a herbaceous spray in 2011. Finally, the intensive herbicide treatment consisted of a site preparation broadleaf spray (2010), a herbaceous spray in 2011, 2012 and 2013, and a broadleaf release spray in 2012 and 2014. We note that the timing, amount, and type of chemicals used were consistent across all study blocks within each treatment; such details are provided elsewhere (Betts *et al.*, 2013; Kroll *et al.*, 2017).

This study was conducted as part of a broader investigation quantifying the demographic response of the White-crowned Sparrow to herbicide application intensity. We selected this species because (1) it has experienced long-term declines within Oregon (Sauer *et al.*, 2014), (2) uses early-successional forests for nesting within forested landscapes in the Pacific Northwest (Chilton *et al.*, 1995) and (3) our prior work found this species nested on all stands across all treatment types (Betts *et al.*, 2013). Thus, focusing on the sparrow allowed us to quantify how offspring sex ratio of a declining early-successional species varied across the entire continuum of intensive forest management that was applied in our study.

### Blood sampling of sparrow nestlings

Offspring sex ratio in sexually reproducing animals may be biased at several points in time during the rearing period (Mayr, 1939), including at conception (primary sex ratio), birth/hatching (secondary sex ratio), or at the time of independence (tertiary sex ratio). In this study, we evaluated sex ratio immediately before the time of independence (hereafter offspring sex ratio, unless otherwise noted). From May through July in both years we searched entire stands regularly to locate as many sparrow nests as possible, monitoring nests that were located by researchers every 2–3 days to determine their fate. For nests that survived until the late nestling stage, we took a small blood sample from each nestling in a brood between nestling Day 5–11 (where Day 0 is the day of hatching) to determine sex ratio, with most broods being sampled on nestling Day 6–8 (i.e. 86% of young). We obtained blood samples by pricking the brachial vein with a sterile 26-gauge needle, drawing blood into a 70 μl microhematocrit capillary tube, and then transferring drawn blood into a 2 ml microcentrifuge vial containing 1 ml Longmire buffer for long-term storage. We obtained blood from as many nestlings as possible in each nest; however, in our analysis we only used nests in which we were able to determine sex for all nestlings present at the time of sampling. All animal handling procedures were undertaken after approval by all appropriate institutional, state, and federal authorities.

### Molecular sexing of nestlings

To streamline molecular sexing, we used direct PCR to amplify sex-specific fragments of the CHD-Z and CHD-W genes (Griffiths *et al.*, 1998). For this analysis, we used Phusion® Blood Direct PCR Master Mix (Thermo Fisher Scientific, Waltham, MA, USA), which contains all the necessary reagents for direct PCR except for primers. This master mix also contains gel loading dye, allowing for the transfer of samples directly to gels for electrophoresis following PCR amplification. For reliable and accurate amplification of our target gene fragments from avian blood stored in lysis buffer, we made several modifications to the manufacturer’s suggested protocol to optimize PCR efficiency. The key modifications we undertook included substantial dilution of blood samples in lysis buffer, and adding betaine to reactions to increase yield and specificity of PCR products (Frackman *et al.*, 1998). In addition, although the manufacturer recommends 50 μl reaction volumes, our optimized protocol performed well in 20 μl reaction volumes, reducing the amount of reagent needed (see below).

Blood samples were stored in lysis buffer for 2–3 years from the time they were initially obtained to the time they were assayed. Due to the high DNA content of avian blood, substantial dilution of the blood-lysis solution was necessary to optimize PCR efficiency. For our samples, which contained 70 μl of whole blood in 1 ml Longmire buffer, we diluted 50 μl of the blood-lysis solution in 600 μl of DNAase-free water, and vortex-mixed the dilution until homogenized. We used primer pair P2/P8 to amplify fragments of the CHD-Z and CHD-W genes (Griffiths *et al.*, 1998), and conducted PCR in 96-well format using a 20 μl final reaction volume. Each reaction consisted of 10 μl of 2X Phusion® master mix, 1 μl each of 10 μM P2 (forward) primer and P8 (reverse) primer, 5 μl of 5 M betaine (1 M final concentration; Acros Organics, Morris, NJ, USA), and 0.5 μl of the diluted blood-lysis sample. We centrifuged loaded plates at 1000 rpm for 1 min prior to initiating PCR.

We performed PCR using a T100™ Thermal Cycler (Bio-Rad, Hercules, CA, USA); PCR cycling conditions consisted of an initial denaturation at 98°C for 5 min, followed by 35 cycles of denaturation at 98°C for 5 s, annealing at 62°C for 15 s, and an extension at 72°C for 15 s, and completed with a final extension at 72°C for 1 min. Following PCR, we centrifuged plates at 1000 rpm for 4 min. We then loaded PCR products (10 μl) directly from plates onto 2% agarose gels and ran gels for 2.5 h at 95 V in 1X TAE buffer. We post-stained gels in a 1 μg/ml ethidium bromide in 1X TAE solution for 1 h prior to imaging under ultraviolet light.

To ground-truth our molecular sexing protocol, we obtained blood samples from *n* = 20 adult sparrows concurrently with our sampling of nestlings. Adults were captured using mist-nets placed within the vicinity of their nest, and a small blood sample was taken as described above for nestlings. The sex of adult female sparrows was determined by the presence of a brood patch (*n* = 15), whereas the sex of adult males was determined by the presence of an enlarged cloacal protuberance (*n* = 5).

### Arthropod sampling

As part of a related study (Betts *et al.*, unpublished data) we sampled terrestrial arthropods to estimate the amount of food available to breeding sparrows on each stand. We randomly located three primary sampling points, all of which were located a minimum of 50 m from any edge within a stand. At each point we conducted restricted-area leaf searches during four sampling periods across the breeding season within each year, examining the upper and lower surfaces of plant leaves and stems (Rodenhouse *et al.*, 2003) and recording the length (mm) and taxonomic order for each arthropod observed. We conducted restricted-area leaf searches on eight species of early-successional plants within three functional groups common in our study area: one conifer (Douglas-fir), five broadleaf shrubs (vine maple, oceanspray [*Holodiscus discolor*], salmonberry [*Rubus spectabilis*], beaked hazel [*Corylus cornuta*], and red alder [*Alnus rubra*]), and two ferns (bracken fern [*Pteridium aquilinum*] and western sword fern [*Polystichum munitum*]). To estimate food availability, we measured both length and mass for a subset of the arthropods from collected from our study sites (87 taxa, *n* = 252 total individuals; Betts *et al.*, unpublished data). From these data we constructed a mass*length regression that indicated arthropod mass and length were strongly correlated on a log–log scale (*r*^2^ = 0.778; *P* < 0.001). We then used this mass*length regression equation to calculate a per-stand estimate of arthropod biomass from each individual measured based on its length. We then summed individual biomass estimates for all sampling sessions on each stand for each year for arthropods >4 mm in length that were in taxonomic groupings known or suspected to be food items of sparrows during the breeding season (Chilton *et al.*, 1995, and references therein).

### Statistical analysis

For our analysis we used a mixed linear modeling approach in the SAS v9.4 statistical environment. We used PROC GLIMMIX to model offspring sex ratio (i.e. proportion of male offspring) as a function of experimental treatment (four levels: no-spray control, light, moderate and intensive herbicide application) with year (two levels: 2013, 2014) and a treatment*year interaction as fixed effects; our random effects included block and stand nested within block. Due to the non-independent nature of nests located on the same stand, we used stand-level proportions to model sex ratio. Although we restricted our analysis to broods where all chicks present in a nest were blood-sampled, we constructed separate models for complete broods (i.e. broods in which all eggs laid were ultimately sexed as nestlings) and incomplete broods (i.e. broods in which at least one previously observed egg and/or nestling went missing prior to the time of blood sampling) in the event that sparrows experienced sex-specific mortality during the nestling stage. However, we detected no treatment effect on sex ratio for either brood type (see section Results), so we combined them for additional analysis (hereafter total broods). To evaluate the relationship between sex ratio and food availability, we used PROC GLIMMIX to model offspring sex ratio (i.e. proportion of male offspring) as a function of stand-level arthropod biomass as a fixed effect, with stand nested within block as a random effect. We provide least-squares marginal means and their 95% confidence intervals (CIs), which are taken over the mean of each covariate within the model, and report parameter (*β*) and odds ratio (OR) estimates and their 95% CIs as measures of effect size.

## Results

### Molecular sexing

Our direct PCR approach was 100% accurate when tested on blood samples taken from known-sex adults (*n* = 20) as determined by physical characteristics related to reproductive activity. Male sparrows (ZZ) produced a single band at ~350 bp for the CHD-Z gene, whereas females (ZW) produced two bands at ~350 and ~400 bp for the CHD-Z and CHD-W genes, respectively. Using the protocol described above, we were able to amplify these same sex-specific fragments of the CHD-Z and CHD-W genes and accurately assign sex to 99.9% of the blood samples we tested, even after some samples were stored in lysis buffer for 3 years prior to laboratory analysis.

### Offspring sex ratio

We located 761 sparrow nests during the course of this study, and we successfully assigned sex to all offspring within a brood in *n* = 403 nests (*n* = 1011 individual determinations; range: 2–5 offspring/nest). Of this total, complete brood sex ratio data were obtained from *n* = 63 nests, and incomplete brood sex ratio data were obtained from an additional *n* = 340 nests. The remaining nests (*n* = 358) were unavailable for blood sampling; most of this subset had experienced nest failure caused by predation (86%).

**Figure 1:**
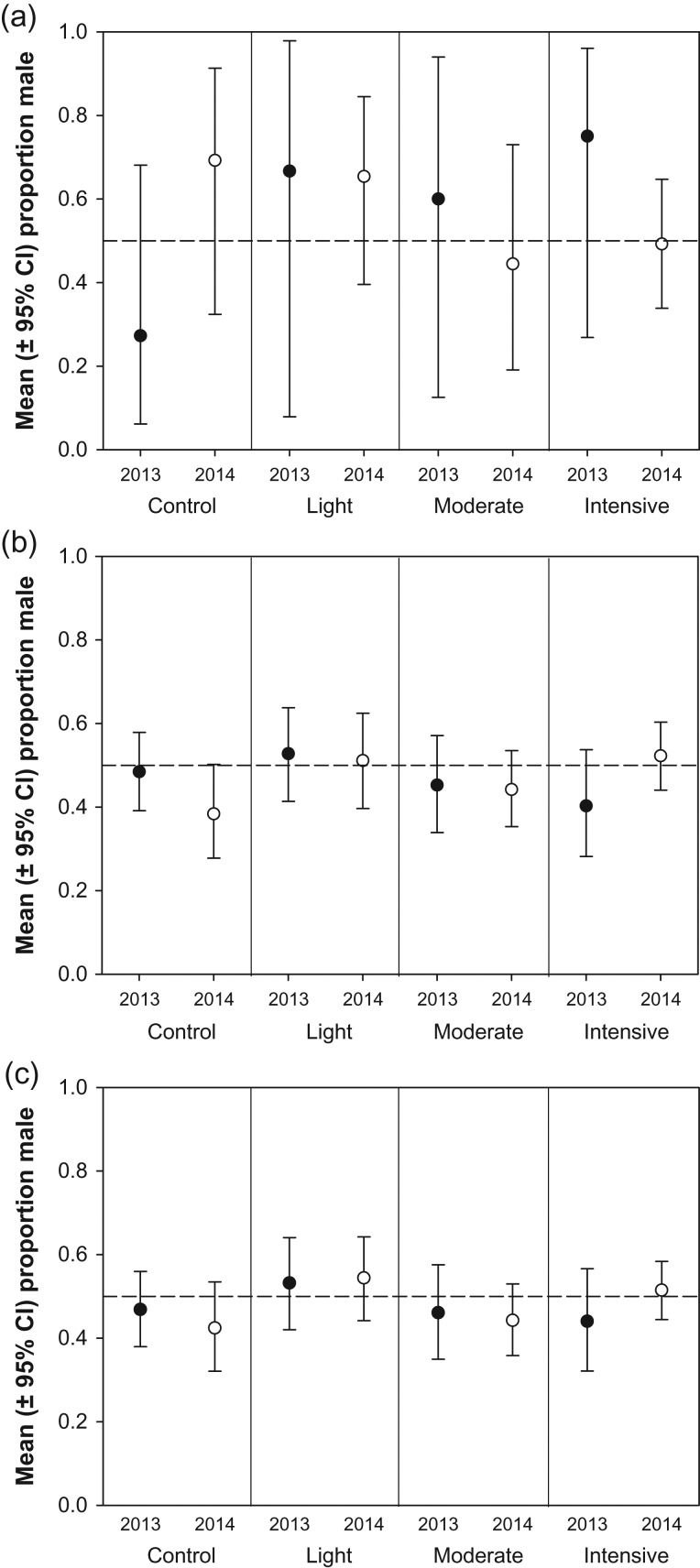
White-crowned Sparrow offspring sex ratio measured prior to fledging in relation to experimental treatment and year for (**a**) complete broods (*n* = 63 nests), (**b**) incomplete broods (*n* = 340 nests) and (**c**) all broods combined (complete + incomplete; *n* = 403 nests). The dashed horizontal line within each panel indicates a 1:1 sex ratio.

When all brood types were considered together, the overall mean offspring sex ratio was 48.3% (*n* = 1011 nestlings in 403 nests). When examining treatment effects in complete sparrow broods, we found no evidence that offspring sex ratio was influenced by either herbicide treatment (*F*_3,5_ = 0.50, *P* = 0.700) or year (*F*_1,5_ = 0.00, *P* = 0.991), with no herbicide treatment*year interaction (*F*_3,5_ = 2.01, *P* = 0.231; Fig. 1a). All parameter estimates for each herbicide treatment overlapped with zero, indicating no evidence for an effect on sex ratio (Table 1). Similarly, we did not detect an effect of either herbicide treatment (*F*_3,12_ = 1.07, *P* = 0.400) or year (*F*_1,12_ = 0.00, *P* = 0.952), with no herbicide treatment*year interaction (*F*_3,12_ = 1.66, *P* = 0.229; Fig. 1b) when examining incomplete broods. Parameter estimates for experimental treatments were less variable in incomplete broods than complete broods, although all overlapped zero (Table 1).

**Table 1:** Parameter estimates (*β*) and associated 95% confidence intervals (CI) for White-crowned Sparrow offspring sex ratio as a function of experimental treatments for complete broods (*n* = 63 nests), incomplete broods (*n* = 340 nests) and all broods combined (*n* = 403 nests)

Level of analysis	Treatment	*β*	95% CI	*P*
Complete broods	No-spray control	−0.09	−1.25, 1.08	0.859
	Light	0.66	−1.00, 2.33	0.351
	Moderate	0.09	−1.23, 1.41	0.866
	Intensive	0.53	−0.56, 1.63	0.266
Incomplete broods	No-spray control	−0.27	−0.57, 0.04	0.082
	Light	0.08	−0.25, 0.40	0.613
	Moderate	−0.21	−0.52, 0.09	0.154
	Intensive	−0.15	−0.47, 0.17	0.320
All broods	No-spray control	−0.22	−0.50, 0.07	0.128
	Light	0.15	−0.15, 0.46	0.298
	Moderate	−0.19	−0.49, 0.10	0.170
	Intensive	−0.09	−0.38, 0.20	0.504

**Figure 2:**
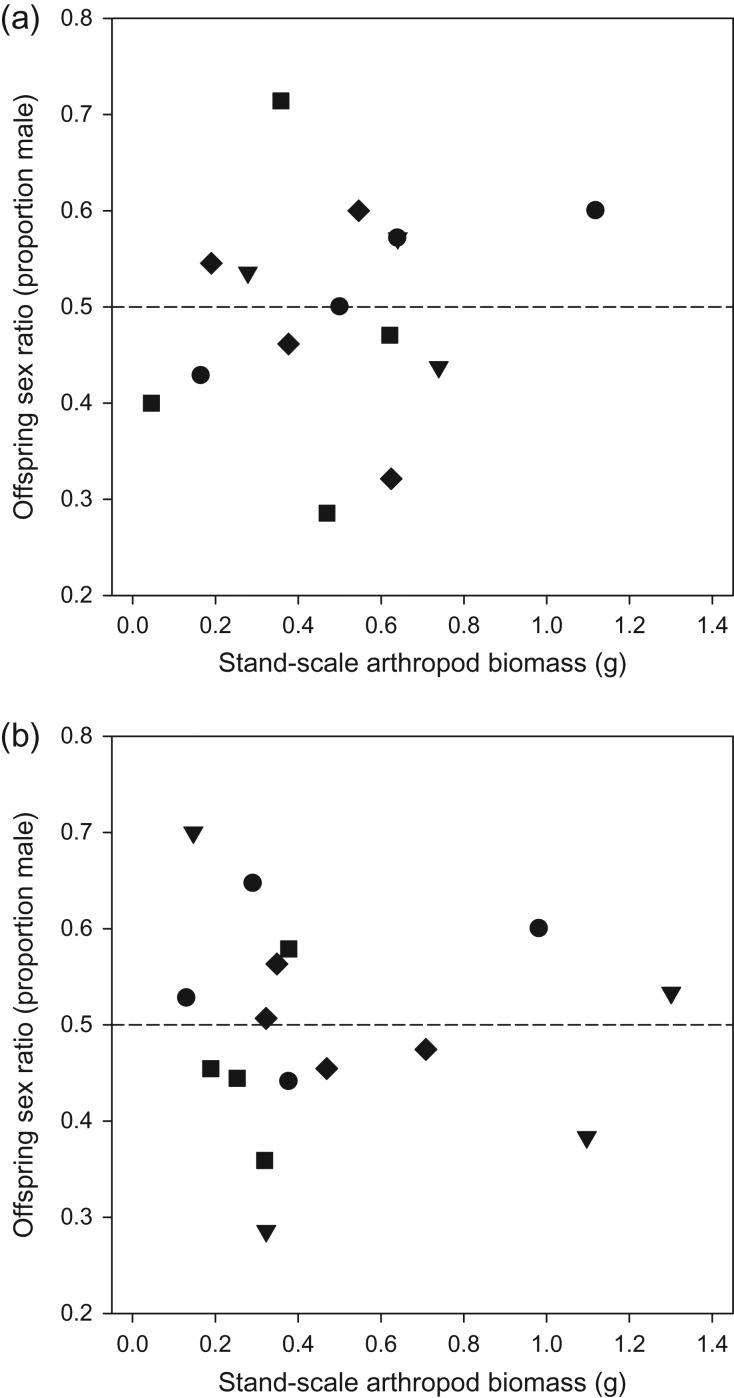
White-crowned Sparrow offspring sex ratio as a function of stand-scale arthropod biomass for the (**a**) 2013 and (**b**) 2014 breeding seasons. Note one stand in 2013 was an outlier and removed because its biomass estimates were >4× greater than any other stand, resulting in *n* = 15 stands assessed in 2013 and *n* = 16 stands assessed in 2014. Triangles represent no-spray control treatments, circles represent light herbicide treatments, squares indicate moderate herbicide treatments, and diamonds indicate intensive herbicide treatments. The dashed horizontal line within each panel indicates a 1:1 sex ratio.

When we conducted our analysis on total broods we again found that sex ratio was not influenced by either herbicide treatment (*F*_3,12_ = 1.51, *P* = 0.263) or year (*F*_1,12_ = 0.03, *P* = 0.865), with no herbicide treatment*year interaction (*F*_3,12_ = 0.60, *P* = 0.625; Fig. 1c, Table 1). For example, the proportion of males in the most intensive herbicide treatment was only 3% greater than in the control, and the odds ratio comparing these two treatments was narrowly bounded and overlapped with 1.0 (OR = 0.88, 95% CI: 0.59, 1.33). We also found no evidence that offspring sex ratio was influenced by stand-level arthropod biomass (*β* = −0.07 [−0.72, 0.57], *F*_1,14_ = 0.2, *P* = 0.643), and arthropod biomass levels fluctuated markedly among individual stands over both years (Fig. 2).

## Discussion

Our study provides the most extensive test to date of whether forest herbicides influence offspring sex ratio in songbirds. Despite this, we found no evidence that sparrow nestling sex ratio was influenced by experimental herbicide application intensity; indeed, we detected only a 3% difference in sex ratio estimates between the most extreme treatments when all broods were considered. This is noteworthy because theory predicts that offspring sex ratio becomes increasingly female-biased as habitat quality is reduced (Trivers and Willard, 1973; Charnov, 1982), and herbicide application led to pronounced and measurable effects on the composition of vegetation on our study sites (Kroll *et al.*, 2017). Why experimental treatments strongly altered sparrow habitat yet did not influence offspring sex ratio is unclear, but one explanation is that female sparrows were unable to control offspring sex (Pike and Petrie, 2003). Although our results are consistent with this hypothesis, previous experimental work with sparrows has found that females are capable of biasing sex ratio at the time of laying (Bonier *et al.*, 2007). As noted above, changes in offspring sex ratio may be an adaptive process by which parents skew offspring sex ratio in a manner that enhances their fitness relative to environmental conditions (Trivers and Willard, 1973; Charnov, 1982). It is therefore possible that, at our study sites, no net fitness benefits would have accrued to females for biasing offspring ratio toward a particular sex (Komdeur, 2012), although we were unable to determine individual fitness measures for females in our study.

The timing of herbicide application on our study sites provided us with an opportunity to examine the potential for a direct influence of herbicide application on offspring sex ratio. Stands in the intensive treatment received herbicide applications during both years of our study (i.e. herbaceous spray in 2013, and broadleaf spray in 2014), whereas stands representing other treatments did not (Kroll *et al.*, 2017). Intensive treatment stands were sprayed in late April and early May and therefore coincided with the timing of territory establishment by sparrows on those sites (J. Rivers, unpublished data). That offspring sex ratio on intensive treatment stands was not found to differ from any of the other treatments suggests spraying of stands in the early breeding season did not affect sparrow offspring sex ratio. It is important to note, however, that our study was not designed *a priori* to distinguish between direct and indirect effects, making us unable to rule out alternative hypotheses. Nevertheless, when considered with the finding that the intensive stands experienced greater reduction in the woody vegetation upon which sparrows forage during breeding (Kroll *et al.*, 2017), our study provides evidence that rules out both direct and indirect effects of herbicide application on offspring sex offspring. We note that species can diverge in their response to herbicides and that the number of studies on herbicide application intensity on wild, free-ranging animal populations is limited; therefore, this topics merits additional study (Kohler and Triebskorn, 2013).

In our study, we also found no support for the hypothesis that herbicide application intensity was linked to a reduction in food available to sparrows during their nesting season. This is consistent with our observations that under-developed nestlings were rare in the >750 sparrow nests we monitored, and that several females successfully raised multiple broods during a single breeding season (Rivers et al., unpublished data). Thus, it appears that the conditions of nutritional stress that can underlie changes in offspring sex ratio (e.g. Myer, 1978) may have been lacking in our study system, potentially reducing the likelihood that female sparrows would adaptively adjust offspring sex ratio. Our previous work has detected a marked change in the density of sparrows on our study sites across time (Betts *et al.* 2013), and current research is focused on evaluating how demographic processes (e.g. nest and juvenile survival) changes in response to experimental treatments and over time (Rivers *et al.*, in review). That work has also found that the consequences of herbicide application intensity on sparrow demography was not as strong as predicted, so it may be that herbicides may minimal effects on sparrow populations in the managed forest landscapes of western Oregon. Additional study of demographic processes of other early-successional forest songbirds, particularly those species that have experienced strong historic declines (Betts *et al.* 2010, 2013), will help establish the extent to which individual species differ in their response to herbicides within the early-successional forest community.

The results of our study are noteworthy given that Leshyk *et al.* (2012) found strong evidence for an effect of timber harvest intensity on offspring sex ratio in the Ovenbird (*Seiurus aurocapilla*). Ovenbird offspring sex ratio was increasingly male-biased as harvest intensity increased (Leshyk *et al.*, 2012), a result that runs counter to theoretical predictions (Trivers and Willard, 1973) and was hypothesized to be driven by sex-specific mortality. Regardless of the mechanism responsible for this result, several features separate the sparrows in our study from Ovenbirds. Although both species are forest-dwelling songbirds, Ovenbirds do not nest in regenerating forest (Porneluzi *et al.*, 2011) and are sensitive to anthropogenic-mediated habitat changes (King *et al.* 1996; Betts *et al.* 2006; Leshyk *et al.*, 2012), both of which may have contributed to the divergent responses found.

In addition to testing of how forest herbicides are linked to offspring sex ratio, our study confirmed that direct PCR is a rapid, accurate, and cost-effective method for sexing birds and is well-suited for studies that require sex determination for a large pool of samples. Based on our experience, we estimate that the approach used in our study was twice as fast and cost half as much as traditional PCR techniques that require purified DNA for analysis. Performing direct PCR from blood eliminated the financial expense and processing time associated with DNA extraction, and omitting this extraction step also reduced opportunities for pipetting error and sample contamination (Swaran, 2014). Moreover, our protocol used a commercially available master mix that contains nearly all the reagents for direct PCR in proper quantities, including gel loading dye, which further streamlined the analysis. The procedure’s relative simplicity also makes it a tractable option for researchers, technicians, and students with limited molecular expertise. Additionally, our approach could use blood stored in lysis buffer for 2–3 years, which negated the need to perform an additional cell-lysing step prior to PCR. This further reduced processing time, but it also demonstrated our analysis technique is compatible with a sample storage medium that is popular with researchers because it eliminates DNA degradation in the field, and allows for samples to be stored at room temperature for extended periods (Longmire *et al.*, 1988). Despite its clear potential to streamline molecular sexing, it is surprising that direct PCR has seen limited application in avian field studies (Morinha *et al.*, 2012), as this technique has been used to sex birds via analysis of whole blood (Dhanasekaran *et al.*, 2016), dried blood spots (Quintana *et al.*, 2009; Suriyaphol *et al.*, 2014; Dhanasekaran *et al.*, 2016), and blood stored in ethanol (Tomasulo *et al.*, 2002). Thus, our study provides an important step forward with a streamlined method for sexing nestlings and adults in sexually monomorphic species, and it is broadly applicable for a wide range of research topics given the pervasiveness of sex-related differences in physiology (Arnold and Itoh, 2011), behavior (Balthazart and Ball, 1995), and life history (Clutton-Brock and Isvaran, 2007; Székely *et al.*, 2014).

Our study used gel electrophoresis to separate and size direct PCR products because these are readily available to most researchers and provide reduced cost for avian sexing procedures. More advanced techniques, such as capillary electrophoresis combined with automated scoring software, can provide faster fragment analysis with higher resolution and increase the speed and efficiency of direct PCR for high-throughput molecular sexing. These advanced approaches are generally more expensive and less accessible than gel electrophoresis due to the need for sequencing equipment and analytic software, but they may be the preferred option for those aiming to optimize speed over cost-effectiveness. Additionally, high resolution approaches (e.g. capillary electrophoresis) may be necessary for molecular sexing in species (e.g. auklets; Dawson *et al.*, 2001) where the sizes of CHD-Z and CHD-W amplicons are too similar to be distinguished effectively by gel electrophoresis.

Although variation in sex ratio has been widely studied in birds (Pike and Petrie, 2003), most studies have focused on testing theory, with little attention paid to evaluating how sex ratio variation in birds may be driven by anthropogenic-based agents such as contemporary forest management practices. Furthermore, we have much to learn about the proximate mechanisms underlying both adaptive and non-adaptive adjustments in avian offspring sex ratio (Pike and Petrie, 2003; Rutkowska and Badyaev, 2008; Szász *et al.*, 2012; Goerlich-Jansson *et al.*, 2013). Thus, a better understanding of the physiological basis of sex ratio modification will shed light on how natural and anthropogenic factors can shape bird populations. Our demonstration that direct PCR provides accurate sexing of blood samples more efficiently and at a lower cost than traditional methods should encourage more researchers to undertake additional investigation of this topic, particularly because such techniques are easily integrated into existing studies examining how land management activities are linked to reproductive output.
